# Transcriptional and Metabolomic Analyses Indicate that Cell Wall Properties are Associated with Drought Tolerance in *Brachypodium distachyon*

**DOI:** 10.3390/ijms20071758

**Published:** 2019-04-10

**Authors:** Ingo Lenk, Lorraine H.C. Fisher, Martin Vickers, Aderemi Akinyemi, Thomas Didion, Martin Swain, Christian Sig Jensen, Luis A.J. Mur, Maurice Bosch

**Affiliations:** 1 DLF Seeds A/S, Højerupvej 31, 4660 Store Heddinge, Denmark; il@dlf.dk (I.L.); tdi@dlf.dk (T.D.); csj@dlf.com (C.S.J.); 2 Institute of Biological, Environmental & Rural Sciences (IBERS), Aberystwyth University, Aberystwyth SY23 3EE, UK; L.Fisher@greenwich.ac.uk (L.H.C.F.); martinj.vickers@gmail.com (M.V.); remiakinyemi@yahoo.com (A.A.); mts11@aber.ac.uk (M.S.)

**Keywords:** *Brachypodium distachyon*, cell wall, drought, FIE-MS, mass spectroscopy, metabolite profiling, RNA-seq, transcriptome profiling

## Abstract

*Brachypodium distachyon* is an established model for drought tolerance. We previously identified accessions exhibiting high tolerance, susceptibility and intermediate tolerance to drought; respectively, ABR8, KOZ1 and ABR4. Transcriptomics and metabolomic approaches were used to define tolerance mechanisms. Transcriptional analyses suggested relatively few drought responsive genes in ABR8 compared to KOZ1. Linking these to gene ontology (GO) terms indicated enrichment for “regulated stress response”, “plant cell wall” and “oxidative stress” associated genes. Further, tolerance correlated with pre-existing differences in cell wall-associated gene expression including glycoside hydrolases, pectin methylesterases, expansins and a pectin acetylesterase. Metabolomic assessments of the same samples also indicated few significant changes in ABR8 with drought. Instead, pre-existing differences in the cell wall-associated metabolites correlated with drought tolerance. Although other features, e.g., jasmonate signaling were suggested in our study, cell wall-focused events appeared to be predominant. Our data suggests two different modes through which the cell wall could confer drought tolerance: (i) An active response mode linked to stress induced changes in cell wall features, and (ii) an intrinsic mode where innate differences in cell wall composition and architecture are important. Both modes seem to contribute to ABR8 drought tolerance. Identification of the exact mechanisms through which the cell wall confers drought tolerance will be important in order to inform development of drought tolerant crops.

## 1. Introduction

The world population is projected to reach 8.6 billion in 2030 and 9.8 billion in 2050 [1,2], which will increase global agricultural crop demand in 2050 by between 60–110% when compared to 2005/2007 levels [3,4]. This increased agricultural production will put strains on associated rising water demands which currently already accounts for ~70% of the world’s extracted fresh water [5]. Future vulnerabilities in fresh water supply are likely to be further aggravated by climate variability with droughts becoming more extensive [6,7]. As a consequence, the development of drought tolerant crops has become one of the global research priorities.

Drought triggers alterations in plant development, metabolism, and gene expression that affect crop yield. In response, plants can use multiple strategies to counter drought via morphological and physiological changes, acting through diverse signaling cascades that can lead to osmotic adjustment [8]. Key features include the involvement of abscisic acid (ABA) and differential gene expression linked to late embryogenesis abundant (LEA) proteins, chaperones and enzymes linked to reactive oxygen scavenging and ion homeostasis, reviewed in [9]. Key transcription factors regulating drought-responsive genes include MYB, MYC, DREB/CBF, ABF/AREB, NAC, and WRKY transcription factors [10,11,12]. Recent studies have revealed some of the crosstalk mechanisms between drought induced signaling networks. For instance, the drought induced transcription factor RD26 (responsive to desiccation 26) was shown to mediate crosstalk between drought and brassinosteroid (BR) signaling [13] while molecular components that integrate the ABA and cytokinin signaling pathways in response to drought stress were also identified [14]. Other recent advances include the identification of a network that triggers a metabolic flux conversion from glycolysis into acetate synthesis to stimulate the jasmonate (JA) signaling pathway to confer drought tolerance [15] and the overexpression of BRL3, a vascular enriched member of the BR receptor family, can confer drought tolerance without penalizing growth [16].

Although many such genes and signaling pathways involved in drought responses and tolerance have been identified in model plants and crops, the biochemical and molecular basis of drought perception and stress adaptation remains largely unclear [17]. Further, domestication has reduced the genetic diversity at certain loci in modern crops, limiting to some extent their potential for developing novel varieties with improved traits [18]. Therefore, mining the genetic and phenotypic diversity present in landraces and wild relatives can provide insights that could accelerate the development of new crop varieties with enhanced drought tolerance [18].

The annual grass *Brachypodium distachyon* (Brachypodium) contains many of the desirable properties (e.g., small genome, short life cycle, and easy cultivation and transformation) required for a model system for the monocotyledon grasses [19]. In addition, a considerable number of genetic and genomic resources have been developed [20,21], including the genome sequences of a large number of accessions [22,23]. Such have positioned Brachypodium as a useful model species to accelerate trait improvement in cereals, forages and biomass crops [24,25,26].

Brachypodium occurs naturally in the circum-Mediterranean region, and grows in a range of geographies and ecological niches, ranging from very dry to moderately moist habitats [21,27]. Thus, Brachypodium is an attractive system for the study of natural variation, with a large collection of inbred lines from geographically diverse locations available [21,28,29]. Accessions from this collection have been included in a number of studies aimed at assessing the natural variation in the response of Brachypodium to drought stress [30,31,32]. However, most of the molecular analyses to identify the genetic components involved in drought stress have focused on a single Brachypodium inbred line, Bd21 [33,34,35]. However, surprisingly few genes have been functionally studied for their involvement in regulating drought tolerance in Brachypodium. The enhanced drought tolerance by the down-regulation of *BdBRI1*, encoding for a putative BR receptor, represent one of the few examples, although the derived plants were also dwarfed [36]. Most drought related functional studies of Brachypodium genes have been carried out by overexpression studies in Arabidopsis, e.g., *BdPP2CA6*, a type 2C phosphatase [37] or *Nicotiana tabacum,* e.g., for *BdGF14d*, a member of the 14-3-3 gene family [38], *BdCIPK31*, a calcineurin B-like protein-interacting protein kinase [39], *BdWRKY36*, a WRKY transcription factor [40], and *BdASR1*, encoding an abscisic acid-, stress-, and ripening-induced (ASR) protein [41].

We previously reported on the detailed phenotyping and metabolite profiling of ten Brachypodium lines exposed to drought stress, selected from a drought screen of an expanded germplasm collection of 138 Brachypodium diploid inbred lines [31]. For this study, we selected one genotype for each of the three previously defined drought categories, ABR8 classified as tolerant, ABR4, intermediate, and KOZ1, susceptible. These three Brachypodium genotypes were exposed to drought stress and leaf tissue samples, harvested at different time-points, were used for genome-wide transcriptome profiling and metabolomics to explore differences in drought-tolerance mechanisms. We describe transcriptomic changes linked to tolerance to drought which could be associated with gene ontology (GO) terms associated with the cell wall. Further, pre-existing patterns of gene expression linked to cell wall GOs appeared to be important for tolerance. Metabolomic profiling indicated few biochemical changes in response to drought. However, metabolites which could be linked to the cell wall were likely to be a source of tolerance. We anticipate that targeting of cell wall-related genes that are associated with stress tolerance could be exploited to develop grass crops that are more tolerant to drought.

## 2. Results and Discussion

### 2.1. Selection of Lines for Transcript Profiling

A diverse collection of 138 different Brachypodium genotypes was previously screened for differences in drought tolerance which was followed by phenomic analysis and metabolite profiling of ten selected genotypes [31]. The different sensitivities of Brachypodium genotypes to water deficit provided an opportunity to gain a deeper understanding of the molecular and metabolic adjustments underpinning such differences. We selected three genotypes (ABR8, ABR4, and KOZ1) from this previous study which exhibited different degrees of susceptibility to water-withdrawal. ABR8, collected from a hillside (322 m altitude) in Siena, Italy, consistently ranked as the most tolerant genotype. KOZ1, collected from the highlands (771 m altitude) in Kozlum, Turkey, represented one of the lines most susceptible to drought stress. ABR4, from the highlands (1043 m altitude) in the Huesca region, Spain, showed an intermediate response to drought stress [31]. These three genotypes were exposed to progressive drought and leaf tissue was collected at the start (T0, control (C)), and after four (T1, C and treatment (D)) and eight (T2, C and D) days of the watering treatments. These time-points reflect our aim to identify changes that occurred relatively early during the exposure to drought stress and therefore define some key insights into tolerance mechanisms rather than loss in viability caused by water withdrawal.

### 2.2. Mapping and Differential Gene Expression Analyses

RNA-seq was used to examine global transcriptional changes in the three Brachypodium genotypes subjected to drought stress. For each of the experimental variables (genotype, time-point, and treatment), more than 90% of the reads could be mapped back to the Brachypodium reference sequence (Table S1).

**Figure 1:**
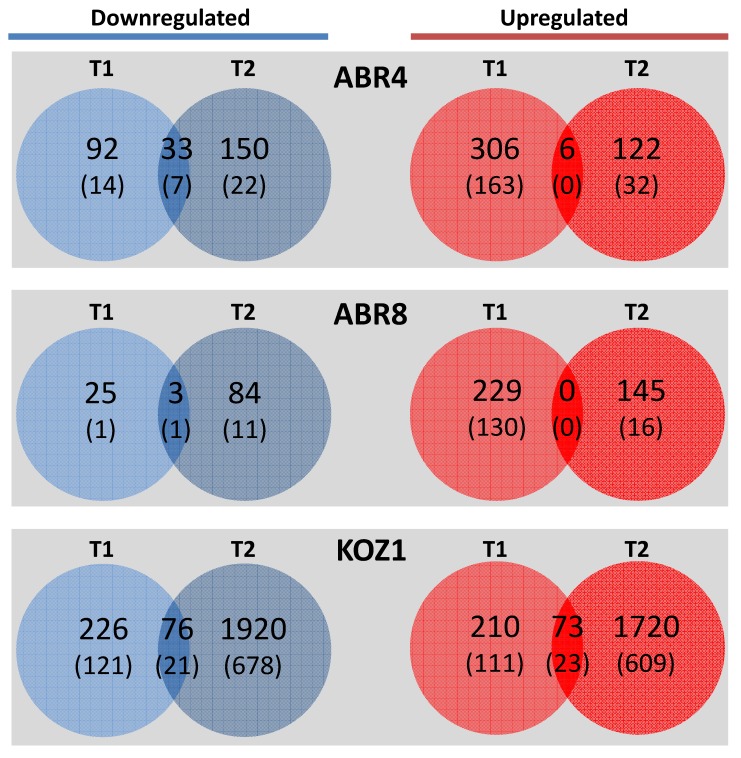
Venn diagram depicting the number of differentially expressed genes (DEGs) in the three Brachypodium genotypes (ABR4, ABR8 and KOZ1) in response to drought stress after 4 days (T1) and 8 days (T2) of withholding water. For each time point the number of genes with > 2-fold change in expression is shown with the number of genes with >4-fold change in brackets.

**Figure 2:**
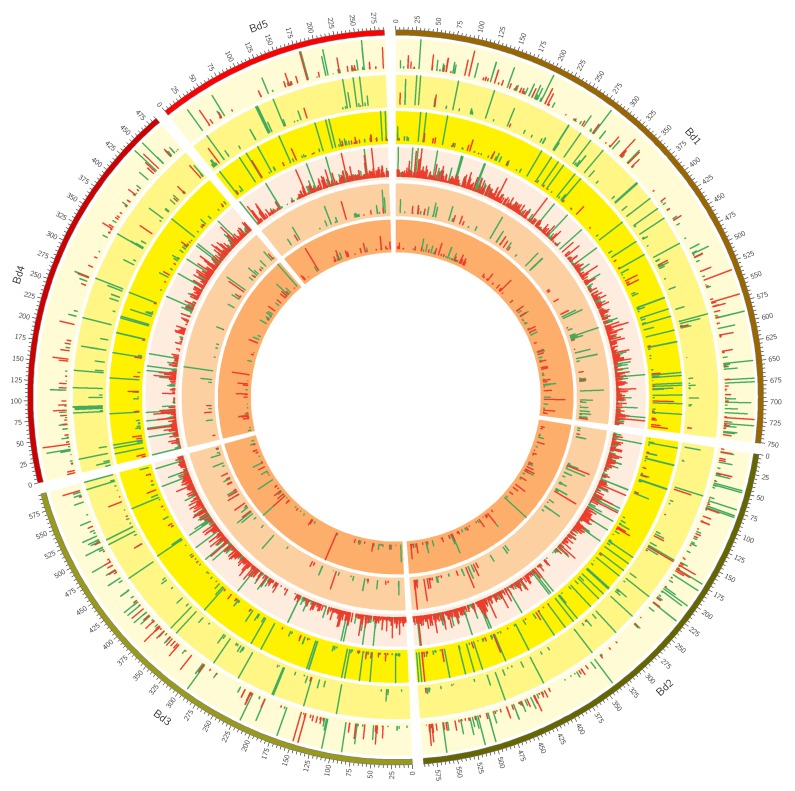
A Circos plot showing the distribution of >2-fold differentially expressed genes (DEGs) over the five Brachypodium n chromosomes (track 1: Bd1-Bd5). Histogram sizes correspond to log2-fold-change with green indicating upregulation and red down-regulation upon drought stress. The three outer yellow rings (tracks 2, 3 and 4) depict DEGs for Brachypodium genotypes KOZ1, ABR8, and ABR4 respectively (outer to inner) in response to drought stress after 4 days (T1). The three inner orange rings (tracks 5, 6 and 7) depict the DEGs for these three genotypes after 8 days (T2) of withholding water.

A total of 4723 transcripts exhibited significant differential expression across the three genotypes when drought treatments were compared against controls for each of the two time-points (≥ two-fold change with *p*-value ≤ 0.05). Leaf samples from drought tolerant ABR8 showed the least number of differentially expressed genes (DEGs), (486), followed by ABR4, (709). KOZ1 exhibited a much higher number of DEGs, 4225 total, most of them (90%) at T2 (3789) (Figure 1 and Figure S1). For both ABR8 and ABR4, the number of up-regulated DEGs decreased at T2 when compared to T1 whilst there was a corresponding increase in the number of down-regulated DEGs from T1 to T2. For KOZ1, the number of DEGs upregulated at T1 was similar to those for ABR8 and ABR4 but there was a substantial number of DEGs that were up- and down-regulated at T2 (Figure 1 and Figure S1). The latter most likely reflected the impact of stress following water withdrawal. The DEGs were distributed across all five Brachypodium chromosomes (Figure 2). The low number of DEGs for ABR8 when exposed to the experimental drought treatment suggests that this genotype possessed innate molecular and physiological mechanisms to tolerate water stress without the requirement for an extensive reprogramming via changes in gene expression.

Upon assigning BRADI gene identifiers to DEGs it became apparent that few genes upregulated at T1 were common to ABR4, ABR8 and KOZ1 (Table S2). In contrast, 31% of ABR4 DEGs downregulated at T1 were also downregulated for KOZ1 at T1 while 26% were also downregulated for KOZ1 at T2. For ABR8, 29% and 42% of the DEGs that were downregulated and upregulated, respectively, at T2 were already down- or upregulated in KOZ1 at T1. This could indicate that part of the drought response is delayed in ABR8 compared to the response in KOZ1. In contrast, a significant proportion of transcripts up- (75%) or down-regulated (49%) at T2 for ABR4 showed a similar pattern for KOZ1 at this time point (Table S2). These data suggest some similarities in the molecular response to drought stress between ABR4 and KOZ1 while the response of ABR8 appeared to be more distinct. This was also suggested by the fact that 31% of DEGs upregulated for ABR8 at T2 were downregulated for ABR4 at T2 when exposed to drought.

### 2.3. Cluster Analyses and GO-Enrichment for Individual Genotypes

**Figure 3:**
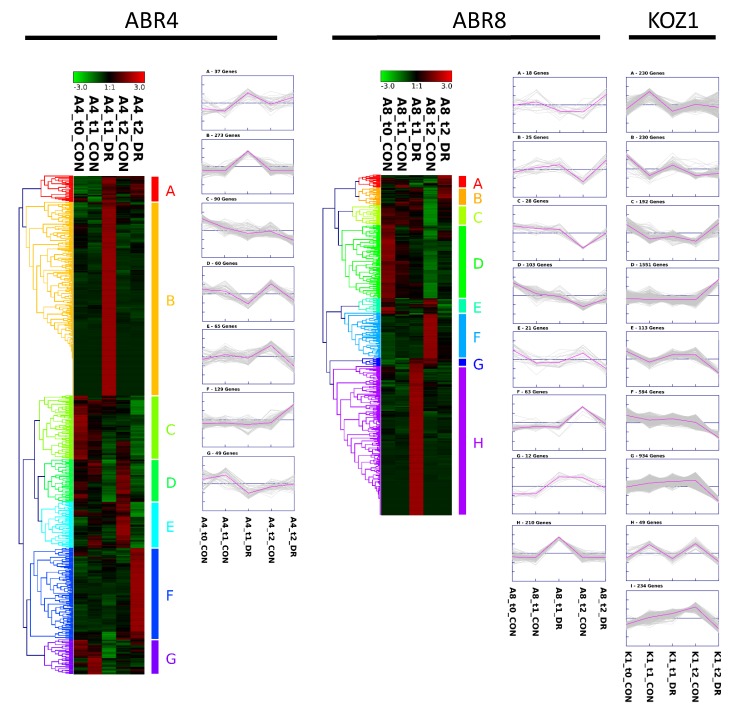
Heat map and cluster results for genes >2 fold differentially expressed between controlled and treated conditions for the three Brachypodium genotypes at either T1 or T2. ABR4: 703 genes; ABR8: 480 genes; KOZ1: 4127 genes. For KOZ1, only the clusters are shown. See Figure S2 for the corresponding KOZ1 heat map. Heat map and clustering was created using complete-linkage hierarchical clustering with the Genesis program [42].

In evaluating the response to water deficit for each of the individual genotypes, genes showing > 2 fold differential expression were visualized by hierarchical cluster analyses (HCA) (Figure 3). Both for ABR4 and ABR8 the largest cluster is formed by genes highly induced by drought stress at T1 (cluster B, 273 genes, and cluster H, 210 genes, respectively). While there is also a cluster of genes upregulated for ABR4 at T2 upon drought stress (cluster F, 129 genes), there are remarkably few genes at T2 that show such pattern for the drought tolerant ABR8 genotype (ABR8 cluster A and B, 43 genes total). Instead, the FPKM (fragments per kilobase of transcript per million mapped reads) value for a large number of genes was either lower (cluster A-D, 174 genes total) or higher (cluster F, 63 genes) for T2 control samples compared with other ABR8 samples. The cluster analysis for KOZ1 is dominated by genes that show either increased (cluster D, 1551 genes) or reduced (cluster E-G, I, 1875 genes) FPKM values at T2 (Figure S2), in agreement with the Venn diagram (Figure 1).

Given the relative magnitude of the transcription response in KOZ1 our enrichment analyses initially focused on that genotype. This indicated 58 different GO terms associated with biological processes significantly enriched for KOZ1 at T1 (Figure S3). Prominent terms included “oxidative stress”, aligning with the known effects of drought stress [43]. Another major GO group was linked to amino-sugar metabolism. Higher glucosamine levels during drought have been linked to increased reactive oxygen species (ROS) production in plants. Indeed, lowering glucosamine content through transgene expression of glucosamine-6-phosphate deaminase (NagB) increased drought tolerance [44]. Many different GO terms (43 in total) were enriched for KOZ1 at T2 while eleven GO terms showed significant depletion (Figure S4), including those associated with “DNA metabolic process” and “RNA processing”. This suggests that DNA replication and repair mechanisms were compromised in this drought sensitive genotype after 8 days of withholding water.

**Figure 4:**
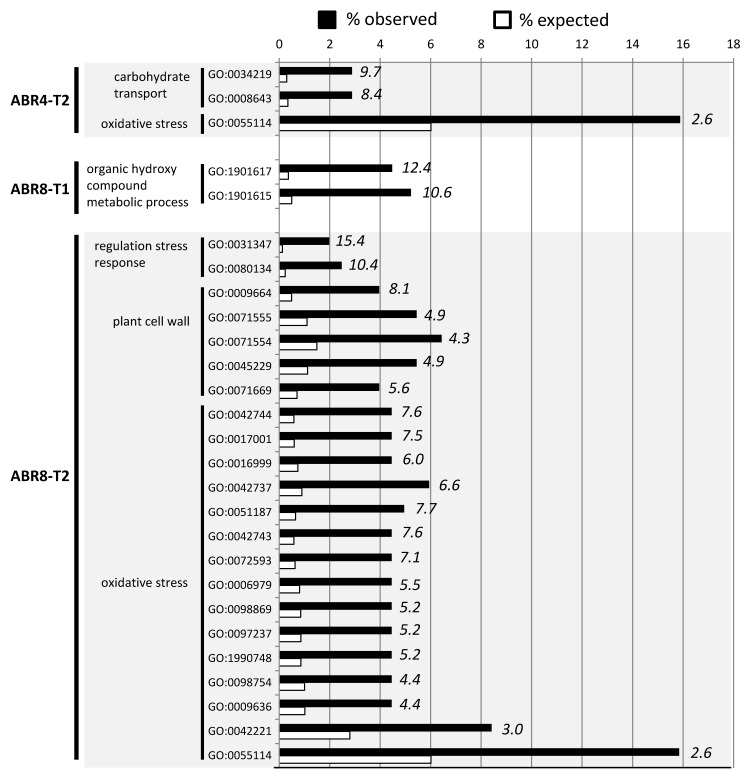
GO term enrichment analysis (*p* < 0.05 FDR corrected) for biological processes of genes >2 fold up or down-regulated in ABR4-T2, ABR8-T1 and ABR8-T2. For each GO term, the expected (white bars) and the observed (black bars) percentage is presented. Numbers in italics indicate the fold enrichment. Only GO-terms with more than 2-fold enrichment are shown. See Figures S3 and S4 for the results of the GO-term enrichment analyses for KOZ1-T1 and KOZ1-T2, respectively.

**Figure 5:**
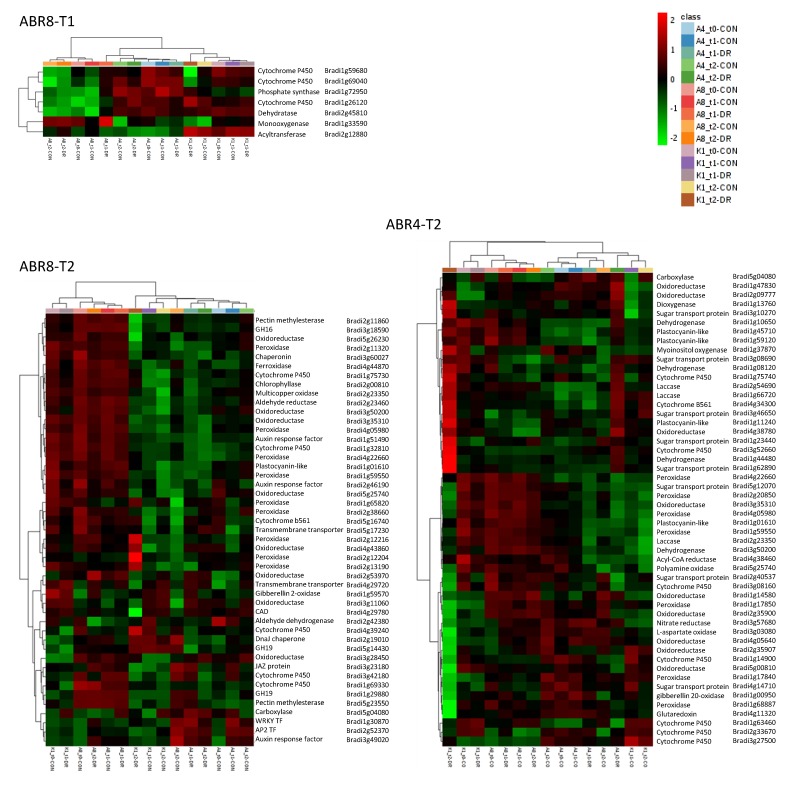
Heat map and cluster results for genes associated with GO enriched terms for ABR8-T1, ABR4-T2 and ABR8-T2. See Tables S3, S4 and S5 for more detailed descriptions of the genes underlying the enriched term for ABR8-T1, ABR4-T2 and ABR8-T2, respectively. Abbreviations: A4, ABR4; A8, ABR8; K1, KOZ1; CON, control; DR, drought; GH, glycoside hydrolase; TF, transcription factor. Heatmap shows normalized FPKM values.

As ABR8 is drought tolerant [31], we were particularly interested in the enriched GO terms for this genotype. At T1, two GO terms associated with “organic hydroxyl compound metabolic process” were significantly enriched in ABR8 (Figure 4); encompassing seven genes (Figure 5; see Table S3 for more details). These were significant as many of these could be tentatively associated in conferring drought tolerance.

Based on the function of the putative Arabidopsis orthologs At3g50660 and At5g36110, three DEGs (Bradi1g69040, Bradi1g59680 and Bradi26120) were potentially involved in brassinosteroid biosynthesis. At3g50660 (*DWF4*) encodes a C-22 hydroxylase that catalyses a rate-limiting step in brassinosteroid biosynthesis whose overexpression in *Brassica napus* led to significantly increased tolerance to dehydration stress [45]. Bradi2g12880 encodes an acyltransferase that may be involved in the deposition of free phytol and free fatty acids in chloroplasts. Its putative Arabidopsis ortholog (At1g54570) maintains the integrity of the photosynthetic membrane during abiotic stress and senescence [46]. Bradi1g3359 was induced > 6 fold compared to untreated control and its Arabidopsis ortholog, At4g12110, encodes a member of the SMO1 family of sterol 4 alpha-methyl oxidases which was previously identified as a cross-species drought-adaptive DEG [47]. Bradi1g72950 is the likely ortholog of Arabidopsis At5g60540/*AtPDX2*; involved in vitamin B6 biosynthesis which increases in response to a range of abiotic stressors including drought [48]. Interestingly, the three genes potentially involved in brassinosteroid biosynthesis and associated with enriched GO-terms for ABR8 at T1, generally showed lower expression in ABR8 compared with ABR4 and KOZ1. Therefore, relatively reduced brassinosteroid accumulation could contribute to drought tolerance as has been noted in some species [49] but not all, e.g., [11].

For ABR8 at T2, enriched GO terms associated with biological processes were perhaps unsurprisingly related to “regulated stress response” (2 GO terms), or “oxidative stress” (15 GO terms) (Figure 4, Table S5). The “oxidative stress” related GO terms were also enriched at T2 in ABR4 (Figure 4, Table S4), making it a feature of all genotypes, and links with drought stress tolerance were therefore not immediately obvious. Of greater interest was the “plant cell wall” (5 GO terms) in ABR8 as we recently linked this cellular compartment with salt stress tolerance in rice [50]. In the rice study, salt tolerance was unequivocally associated with a pectin methyltransferase, whilst in this drought experiment in Brachypodium, pectin methylesterases (PMEs) were targeted (Bradi1g11860 and Bradi5g23550) (Figure 5, Table S5). Pectin modifications, in particular PME-regulated pectin methylesterification may affect stiffness and hydration status of the cell wall matrix during drought stress responses [51]. PMEs have been shown to be involved in cell wall remodeling in response to various abiotic stresses including heat, cold acclimation, UV light, and heavy metals [51]. Further, demethylesterification of pectins in the cell wall of guard cells is important for stomatal function [52], suggesting that PMEs may control water loss under drought conditions. However, our Brachypodium accessions exhibited no significant difference in stomatal conductance with progressive drought treatment [31].

Other cell wall genes included glycoside hydrolase (GH) enzymes; GH16 (Bradi3g18590) and two GH19 (Bradi1g29880 and Bradi5g14430). GHs are involved in orchestrating cellulose–cellulose interactions mediated by xyloglucans and important in regulating cell wall extensibility [53,54] remodeling and reorganization to facilitate plant cell expansion, differentiation, maturation, and wall repair [55]. Most of the cell wall-related DEGs were peroxidases (POX, 8 in total), suggesting an important role in regulating cell wall properties during drought stress. These peroxidases were generally highly expressed in all ABR8 samples (except the T2 control). The highest expression for three of the peroxidases was seen in KOZ1 T2 so it may be that the early expression of POX is advantageous as seen for ABR8.

Two other enriched GO terms for ABR8 at T2 were related to the regulation of stress responses (GO: 0031347 and GO: 0080134). The associated genes encoded two jasmonate ZIM domain (JAZ)-containing proteins (Bradi1g72590 and Bradi3g23180), a WRKY (Bradi1g30870/BdWRKY68) and AP2 domain containing (Bradi2g52370) transcription factor (TF), and a chaperonin (Bradi3g60027). JAZ proteins bind directly to and repress the activity of various transcriptional regulators of jasmonate responses. When intracellular levels of jasmonate rise above a threshold concentration, JAZ proteins are rapidly degraded via the ubiquitin/26S proteasome-dependent proteolytic pathway, releasing the JAZ-bound TFs and activating JA responses [56,57]. Transgenic rice plants overexpressing *OsJAZ1* showed a decrease in tolerance to drought stress [58], while the expression of a cotton JAZ gene, *GaJAZ5*, in Arabidopsis significantly increased their tolerance to water stress conditions [59]. This apparent contradiction possibly reflects the complexity, and cross-talk between, hormone mediated stress responses to drought to balance resource allocation between growth and defense [60]. WRKY and AP2 proteins represent large TF families in plants shown to play critical roles in plant responses to biotic and abiotic stresses, including drought, mostly by regulating plant hormone signal transduction pathways [61,62]. The Arabidopsis orthologue (WRKY46) of Bradi2g44270 (2.3-fold upregulated at T1) regulates genes involved in cellular osmoprotection and redox homeostasis under dehydration stress [63]. Several BdERF TFs belonging to the AP2/EREBP family were found to be induced by drought and salt stress in Brachypodium Bd21 [64]. The three BdERFs identified as DEGs in our study (not associated with GO terms), were all downregulated in ABR8 at T2 (Bradi5g21250, 2.5-fold; Bradi5g25570, 2.2-fold, and Bradi3g18070, 6.2-fold). The Arabidopsis ortholog of the chaperonin Bradi3g60027, At5g20720/CPN20, can mediate the activation of iron superoxide dismutases (FeSODs) in chloroplasts [65], which may provide protection from drought induced oxidative stress. In this context, DEGs associated with enriched GO terms related to oxidative stress for ABR8 at T2 were mostly encoding for oxidoreductase, cytochrome P450 and auxin response factor (ARF) proteins (Figure 5 and Table S5).

### 2.4. Constitutive Expression of Cell Wall Related Genes in ABR8

Genotypic differences between the three Brachypodium genotypes were highlighted in a HCA based on genes showing >2 fold differential expression across all the samples (all of the genotypes and time-points). This identified genotype specific differences in gene expression levels independent of treatment (Figure S5). Cluster A (337 DEGs) and in particular cluster B (351 DEGs) contains genes that are higher expressed in ABR8 and in cluster K predominantly lower compared to the other two genotypes. Cluster N contains transcripts that mostly show higher expression levels in ABR4 compared with ABR8 and KOZ1.

**Figure 6:**
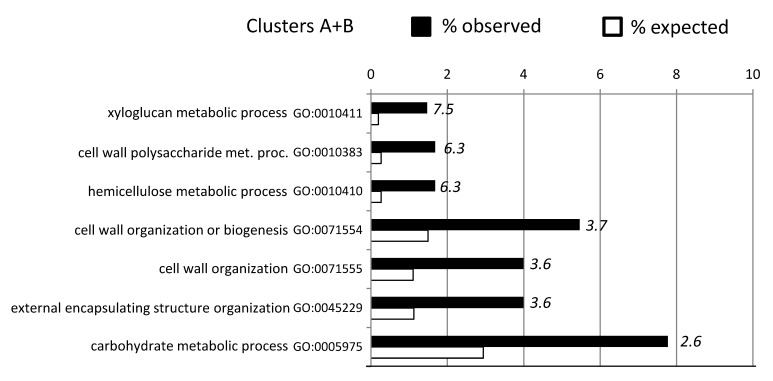
GO term enrichment analysis (*p* < 0.05 FDR corrected) for biological processes of genes in cluster A and B (see Figure S5). For each GO term, the expected (white bars) and the observed (black bars) percentage is presented. Numbers in italics indicate the fold enrichment. Only GO terms with more than 2-fold enrichment are shown.

GO enrichment analyses were performed for clusters A, B and K and while no GO terms were enriched for cluster K, for clusters A and B a number of GO terms were enriched (Figure 6). Notably, these GO terms were all associated with the cell wall; suggesting a possible continuity between the responses to drought and likely innate sources of tolerance. A heat map for the 50 genes in the A + B cluster associated with cell wall-enriched GO terms is shown in Figure S6.

GO terms included genes that encode for xyloglucan endotransglycosylases/hydrolases (XTH), XTHs can modify the cell wall architecture by either cleaving and, often, also re-joining xyloglucan molecules in primary plant cell walls through its xyloglucan endo-transglycosylase (XET) activity, or by the irreversible shortening of xyloglucan chains through its xyloglucan endo-hydrolase (XEH) activity [66,67]. Increased expression levels of XTH genes have been observed in plants exposed to various abiotic stresses [51,68]. The heterologous expression of a drought induced hot pepper (*Capsicum annuum* L.) XTH, *CaXTH3*, in Arabidopsis and tomato (*Solanum lycopersicum*), significantly improved their tolerance to severe water deficit [69,70]. Other genes included two putative xyloglucan fucosyltransferases (GT37), a GT34 xyloglucan 6-xylosyltransferase, and an irregular xylem 15 like protein (IRX15), involved in xylan biosynthesis of plant cell walls. The generally higher expression of XTHs and xylan biosynthesis genes in ABR8 samples when compared with ABR4 and KOZ1 samples (Figure S6), could indicate that relative abundance and remodeling of xyloglucan tethers between the cellulose microfibrils can influence drought tolerance.

Other cell wall linked genes in the A + B cluster included GHs, PMEs, two expansins and a pectin acetylesterase (Table 1; Table S6). Expansins bind to cellulose and mediate acid-induced loosening of plant cell walls without mechanically weakening the cell walls and are often upregulated by abiotic stress conditions. Where genetic approaches have altered expansin expression, this has led to enhanced resistance to abiotic stresses, albeit often accompanied by changes in plant growth and development [71]. For example, heterologous expression of a wheat β-expansin (TaEXPB23) in tobacco improved drought resistance compared with wild type plants [72,73]. It may be that the “misregulation” of expansin is sensed as a disturbance in cell wall integrity, triggering a defense response [71]. Interestingly, TaEXPB23 expression in tobacco improved oxidative stress tolerance and enhanced the cell wall bound POX activity, possibly indicating a relationship between expansin and POX-mediated cell wall crosslinking in the response to stresses [74]. Indeed, POX (6 genes), implicated in both loosening or stiffening of the cell wall under drought stress [68], showed a distinct higher expression in ABR8 samples.

Future work will need to establish if the cell wall properties for ABR8 are indeed distinct compared to those of drought susceptible Brachypodium accessions, particularly as the identified cell wall DEGs for ABR8 mostly relate to enzyme activities that regulate and modify the fine structure of xyloglucan and pectin.

### 2.5. Metabolomics Targets the Cell Wall as an Innate Source of Drought Tolerance in ABR8

**Figure 7:**
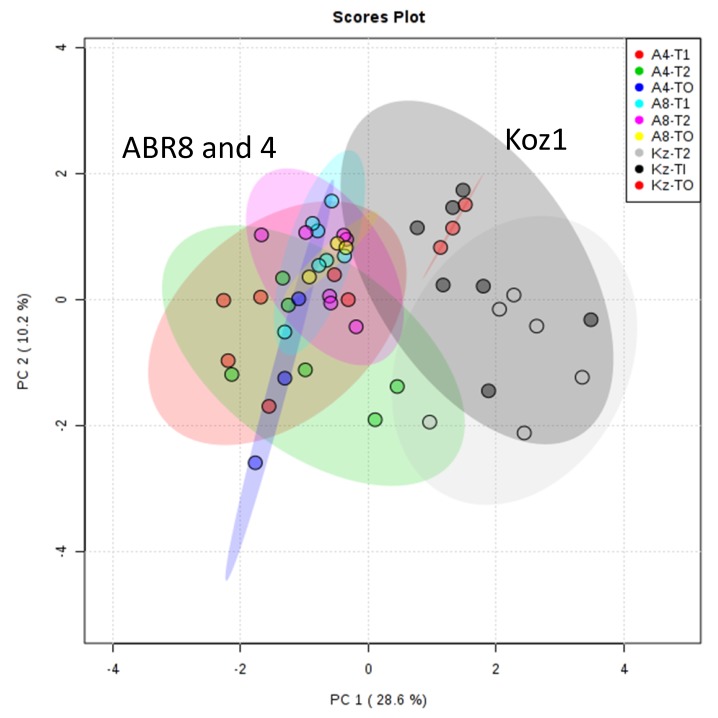
Principal Component Analysis (PCA) of metabolite profiles of the three Brachypodium genotypes (ABR4 [A4], ABR8 [A8] and KOZ1[KZ]) in response to drought stress after 4 days (T1) and 8 days (T2) of withholding water compared to well-watered controls (T0).

To obtain further insights into the possible mechanisms of drought tolerance, samples from the same experiment as used for RNA-seq based transcript profiling were assessed using metabolomic approaches. In this case, since the actual experiment contained six replicates (three of which were used for RNA-seq), all the available replicate samples for T0 (control), T1 (drought) and T2 (drought) were included. Thus, for each of the samples/treatments a minimum of 3 replicates and maximum 6 replicates were used for our metabolomic analyses. As we also demonstrated in [31], adoption of FIE-MS allowed a wide-ranging assessment of the biochemical alterations linked to physiological changes. Principal component analysis (PCA) for all samples, based on a matrix of 2311 m/z x 48 samples (Table S7), suggested no significant differences between ABR8 and ABR4 in both control samples and those following drought treatment (Figure 7). KOZ1 was distinctive, in that it exhibited both genotypic and stress responsive differences in the metabolome. These results align with the major changes observed for the transcriptomic responses of KOZ1 (Figure 1) and the predominance of innate genotypic differences (Figure S5). To avoid focusing on these genotypic differences, the metabolomic responses of each genotype to stress were considered separately (Figure S7). PCA again suggested that only KOZ1 exhibited a wide-ranging response to drought (Figure S7 A, B vs. C). This notwithstanding, ANOVA corrected for false discovery rates (FDR) identified statistically significant (*p* <0.05) changes in each genotype. HCA and heat maps of the significant metabolites suggested that whilst responses to T1 and T2 were difficult to separate, these were clearly different to unstressed controls in each accession.

In an attempt to provide functional information on the responses to drought in each accession, the significant metabolites were assessed for pathway enrichment (Figure S8). In the case of ABR8, no significant pathway enrichment was observed. With ABR4, some pathways were targeted but only glutamate metabolism and the glucose alanine cycle exhibited robust statistical validity with satisfactory Holm P (for multiple testing) and FDR. For KOZ1, only brassinosteroid metabolism could be considered to be enriched. This could reflect the reduced expression of brassinosteroid biosynthesis genes in the ABR8 transcriptome (Figure 5). Indeed, in the heat map of ABR8 (Figure S7A), castasterone, an important brassinosteroid, was elevated only in T0 samples. Given some reports of brassinosteroids conferring drought tolerance; e.g., as demonstrated by the over-expression of its receptor [16]; it could be that the accumulation of a single brassinosteroid is important for drought tolerance. Such a subtle accumulation pattern could be missed in our GO term-based transcriptional analyses.

**Figure 8:**
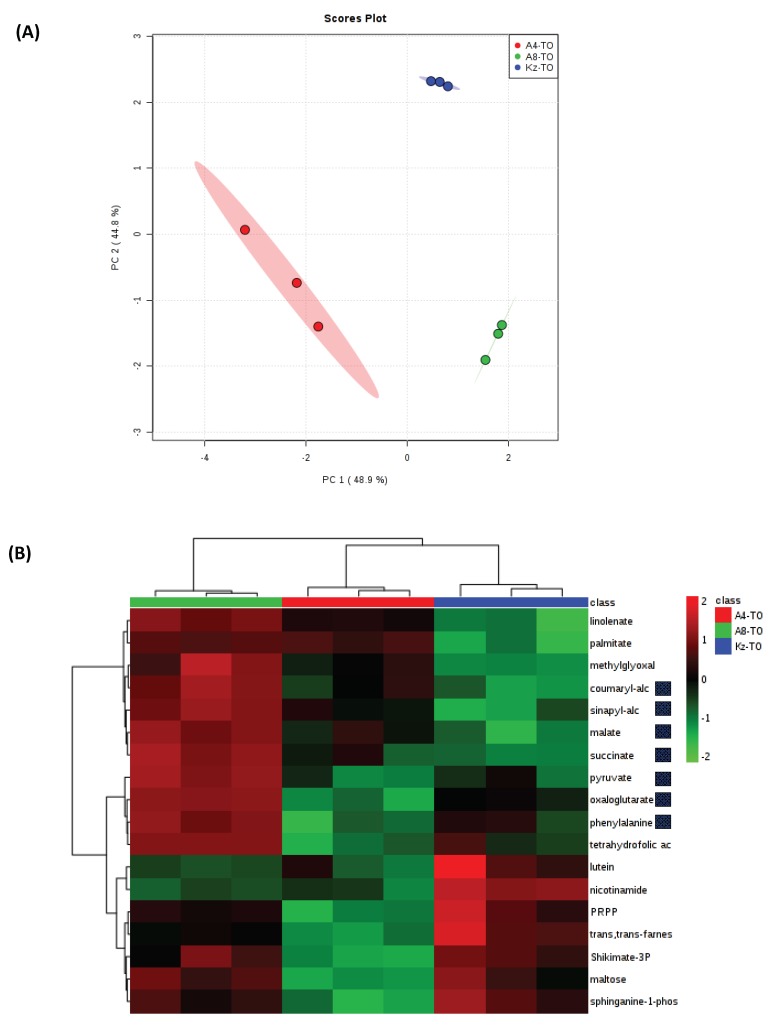
(**A**) Principal Component Analysis of metabolite profiles the three Brachypodium genotypes ABR8 [A8], ABR4 [A4] and KOZ1[KZ]) in well-watered controls (T0). (**B**) Also shown as heat maps are significantly significant metabolite changes (*p* < 0.05; FDR <0.05) for each accession. Metabolites highlighted in yellow are associated with bioenergetic metabolism and in orange, cell walls.

Based on this observation along with the relative lack of a major response to drought in ABR8 and ABR4, we explored innate differences between accessions that could represent sources of tolerance. PCA indicated that each accession had a highly distinctive metabolome from which statistically significant metabolites could be identified (Figure 8). Although it was not possible to demonstrate pathway enrichment based on these metabolites, inspection of those which were increased in ABR8 compared to KOZ1 indicated increases in components of the TCA cycle and components that could be linked to the cell wall (Figure 8B). The latter were the mono-lignols; coumaryl and sinapyl-alcohols and phenylalanine which feed into the phenylpropanoid pathway leading into lignin biosynthesis.

Phenylalanine is metabolized by phenylalanine ammonia lyase (PAL) to feed into phenylpropanoid metabolism. PAL expression has been heavily associated with responses to stress, including drought [75]. Interestingly, PAL suppression in Brachypodium Bd21 did not affect responses to drought [76] but this accession is quite sensitive to drought and a PAL influenced mechanism could be relevant to ABR8. Further, some studies have also associated lignin biosynthesis with drought tolerance in maize [77] and rice [78]. Possible mechanisms through which elevated lignification could confer tolerance to drought stress include improved resistance to drought-associated embolisms in the xylem [79] and influencing stomatal closure [78]. We have not tested embolism formation in our lines but, as we have noted, ABR8 does not display any different stomatal responses compared to KOZ1 [31].

Superficial comparisons of transcriptomic and metabolomic data could suggest that there is little correspondence between the two ‘omes. In particular, phenylpropanoid pathway associated gene expression was not observed as either DEGs or innate differences between accessions. This notwithstanding, it is striking that the same cellular compartment—the cell wall—was indicated by both transcriptomic and metabolomic approaches as a potential innate source of drought tolerance in ABR8. Further, the metabolomic approach that we adopted could not yield insights into complex cell wall carbohydrate polymers. Future analyses will need to focus on how the cell wall, possibly by influencing water loss or cell signaling, is involved in conferring drought tolerance.

## 3. Materials and Methods

### 3.1. Plant Growth and Treatment

Three genotypes of Brachypodium ABR4, ABR8 and KOZ1, were grown in John Innes No 1 potting compost with grit-sand at a 4:1 ratio. Plants were grown under controlled environment conditions in a growth chamber at 21 °C under a 16 h photoperiod with 176 μmol m^−2^ s^−1^ photon flux density supplied by white fluorescent tubes (OSRAM, Garching, Germany). Plants were well-watered and grown for 32 days after germination before a progressive drought treatment was applied. At this point an equal number of well-watered (control) and water-deprived (treated) replicates were organized in a latin square block arrangement. The progressive drought treatment lasted 8 days.

### 3.2. RNA Isolation

For each of the genotypes, leaf tissue was harvested at three time points for RNA extraction; at the start (T0, control (C)), after 4 days (T1, both C and treatment (D)) and after 8 days (T2, both C and D). In total, 45 plants were harvested ((T0 + T1C + T1D + T2C + T2D) × 3 genotypes × 3 biological replicates) and ground to a fine powder in a mortar and pestle with liquid nitrogen. Total RNA was extracted using Trizol reagent (Invitrogen, Carlsbad, CA, USA) and purified using RNeasy MinElute Cleanup Kit columns (Qiagen, Valencia, CA, USA). On-column DNase I digestion (DNase-Free DNase Set, Qiagen) was performed according to manufacturer protocols to eliminate genomic DNA contamination. Following extraction, the quality and quantity of the RNA samples was determined by absorbance measurements at 230, 260, and 280 nm (Nanodrop 1000 spectrophotometer, NanoDrop Technologies). RNA integrity was evaluated on a 0.8% (*w*/*v*) agarose gel. After all samples passed BGI’s (BGI, Shenzhen, China) strict quality control tests criteria, BGI performed the TruSeq library construction and sequencing with Illumina HiSeqTM 2000.

### 3.3. Transcript Assembly and Differential Expression Analysis

The obtained raw data (45 pairs of fastq files) is available in the Gene Expression Omnibus (GEO) (https://www.ncbi.nlm.gov/geo/) with accession number GSE126992. Raw reads were quality trimmed using trim galore version 0.4.1 [80] with the following parameters; --quality 28 --phred64. Transcript assembly and differential expression analysis was performed using the Cufflinks software package version 2.2.1 [2]. The trimmed reads were mapped to the Brachypodium reference genome (version 314 v3.0 from Phytozome, [23]) using tophat (version 2.1.1) and genome annotation version 314 v3.1. Differential expression analysis was performed on the mapped reads, using cuffdiff (version 2.2.1). Table S1 gives an overview of the coverage and sequences pairs with on average more than 93% of the pairs mapped to the genome. The main differential expression data output from cufflinks is summarized by the file “gene_exp.diff”. In this file genes were only used in subsequent analyses if the field “Test status” was equal to “OK” (e.g., the read alignments passed quality controls such as sufficient depth) and the “significance” field was set to “yes” (i.e., the gene’s *p*-value is greater than the FDR after Benjamini–Hochberg correction for multiple-testing). Subsequently, annotations from Phytozome (the file Bdistachyon_314_v3.1.annotation_info.txt) were added to this table, which included GO terms. To identify differentially expressed genes between the control and treated conditions at each time point (T1 and T2) for each sample (ABR4, ABR8 and KOZ1), genes with a log2-converted fold-change with respect to FPKM between > 1 and < -1 were extracted from the Cufflinks results. Clustering of differentially expressed genes showing more than 2-fold differential expression was performed using Genesis [42] version 1.8.1. Each genotype was analyzed separately as well as all the data together. The FPKM values were normalized by gene and the normalized FPKM values were then clustered using hierarchical complete linkage clustering with automatic cluster assignment and automatic threshold both set. GO enrichment was performed using PANTHER [81]. The circos software package was used for visualizing the distribution of the differentially expressed genes [82].

### 3.4. Metabolite Profiling

Powdered leaf samples which were also used for RNA extraction were used to isolate metabolites. Thus 1mL of chloroform: methanol: dH2O (1:2.5:1) was added to 50 μg of powdered samples and shaken at 4 °C degrees for 15 min and returned to ice. Samples were centrifuged at 4 °C degrees at 5000 x ɡ for 3 min and dried in vacuum. Afterwards, 250 µL of 70% methanol was added to the samples and resuspended by vortexing for 5 s. For flow infusion electrospray ionization high resolution mass spectrometry (FIE-HRMS), 100 µL of each sample was transferred into a glass vail and sealed. All samples were run in duplicate with no significant differences in the results obtained. Flow infusion electrospray ionization high resolution mass spectrometry (FIE-HRMS) was performed using Q executive plus mass analyzer instrument with UHPLC system (Thermo Fisher Scientific©, Bremen, Germany), where m/z were generated in positive and negative ionization mode in a single run as described by [83]. The metabolomics profiles were investigated using the R-based platform Metaboanalyst 4.0.

## 4. Conclusions

Plant cell walls are highly complex and dynamic structures that, besides providing mechanical support and supporting growth, need to respond to various environmental and developmental cues and fulfil important functions in the defence against biotic and abiotic stresses [51,84,85]. Our transcriptional and metabolomic analyses have suggested that cell wall features in Brachypodium could be a source of drought tolerance and implicate two different modes: (i) An active response mode linked to stress induced changes in cell wall features, and (ii) an intrinsic mode where innate differences in cell wall composition and architecture are important. Both these modes seem to contribute to the drought tolerance of ABR8. These findings should provide a platform for future studies aimed at identifying drought-induced changes in cell wall composition and architecture, an area for which we currently have little knowledge. More specifically, we need to understand the mechanisms by which cell wall peroxidases, pectin modifications, xylan biosynthesis, and glycoside hydrolases such as XTHs, influence cell wall properties, including its hydration status, in response to drought, and the cell wall integrity sensing mechanisms that trigger such drought-induced adaptive changes in cell wall architecture. Identification of the exact mechanisms through which the cell wall confers drought tolerance will be important in order to inform development of drought tolerant crops.

## Figures and Tables

**Table 1:** Overview of genes associated with enriched GO-terms cluster A + B.

Gene Category	Count	Gene ID’s			
GH1	1	Bradi5g15527			
GH3	2	Bradi4g20197	Bradi5g23470		
GH10	1	Bradi1g12710			
GH13	1	Bradi5g00540			
GH16	4	Bradi3g52307	Bradi1g33840	Bradi1g27867	Bradi3g18590
GH17	3	Bradi2g60497	Bradi3g07385	Bradi3g33277	
GH18	1	Bradi2g55630			
GH19	1	Bradi1g29880			
GH28	2	Bradi2g18030	Bradi1g76890		
GH29	1	Bradi4g35500			
GH32	1	Bradi4g07847			
GH35	1	Bradi1g37450			
GT4	1	Bradi1g20890			
GT8	1	Bradi2g48710			
GT28	1	Bradi2g13780			
GT34	1	Bradi1g64560			
GT37	2	Bradi3g10230	Bradi3g58040		
GT65	1	Bradi1g43560			
GT77	1	Bradi1g76460			
peroxidase	6	Bradi2g20850	Bradi4g05980	Bradi1g59550	Bradi2g11320
		Bradi2g04490	Bradi2g37000		
PME	2	Bradi2g11860	Bradi5g23550		
expansin	2	Bradi2g53580	Bradi2g22290		
Others	13	Bradi3g16307	Bradi1g20270	Bradi4g05860	Bradi1g61630
		Bradi3g04460	Bradi3g16927	Bradi4g34430	Bradi4g18170
		Bradi3g53830	Bradi5g12260	Bradi3g50810	Bradi2g45320
		Bradi4g44860			

Abbreviations: GH, glycoside hydrolase; GT, glycosyl transferase; PME, pectin methylesterase.

## Supplementary Materials

Supplementary materials can be found at https://www.mdpi.com/1422-0067/20/7/1758/s1.
