# Supplementary material for: Transcriptional and Metabolomic Analyses Indicate that Cell Wall Properties are Associated with Drought Tolerance in Brachypodium distachyon
Source: Int J Mol Sci. 2019 Apr 10;20(7):1758. doi: 10.3390/ijms20071758 (PMC6479473; doi:10.3390/ijms20071758)
Supplement: Supplementary file 1 [file ijms-20-01758-s001.zip › supplementary Figures 1-8.pdf]

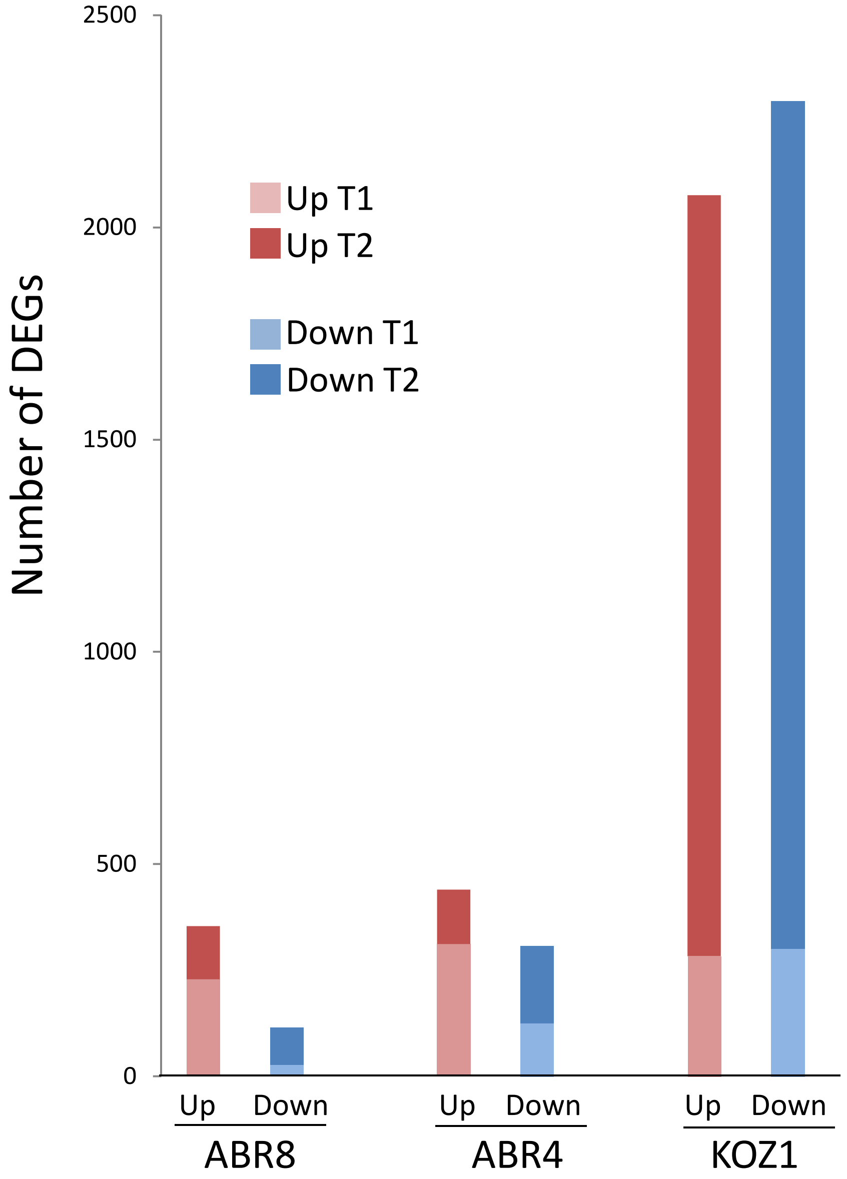

Figure S1. Bar graph depicting the number of differentially expressed genes (DEGs) in the three *Brachypodium distachyon* genotypes (ABR4, ABR8 and KOZ1) in response to drought stress after 4 days (T1) and 8 days (T2) of witholding water. For each time point the number of genes with > 2-fold change in expression is shown.

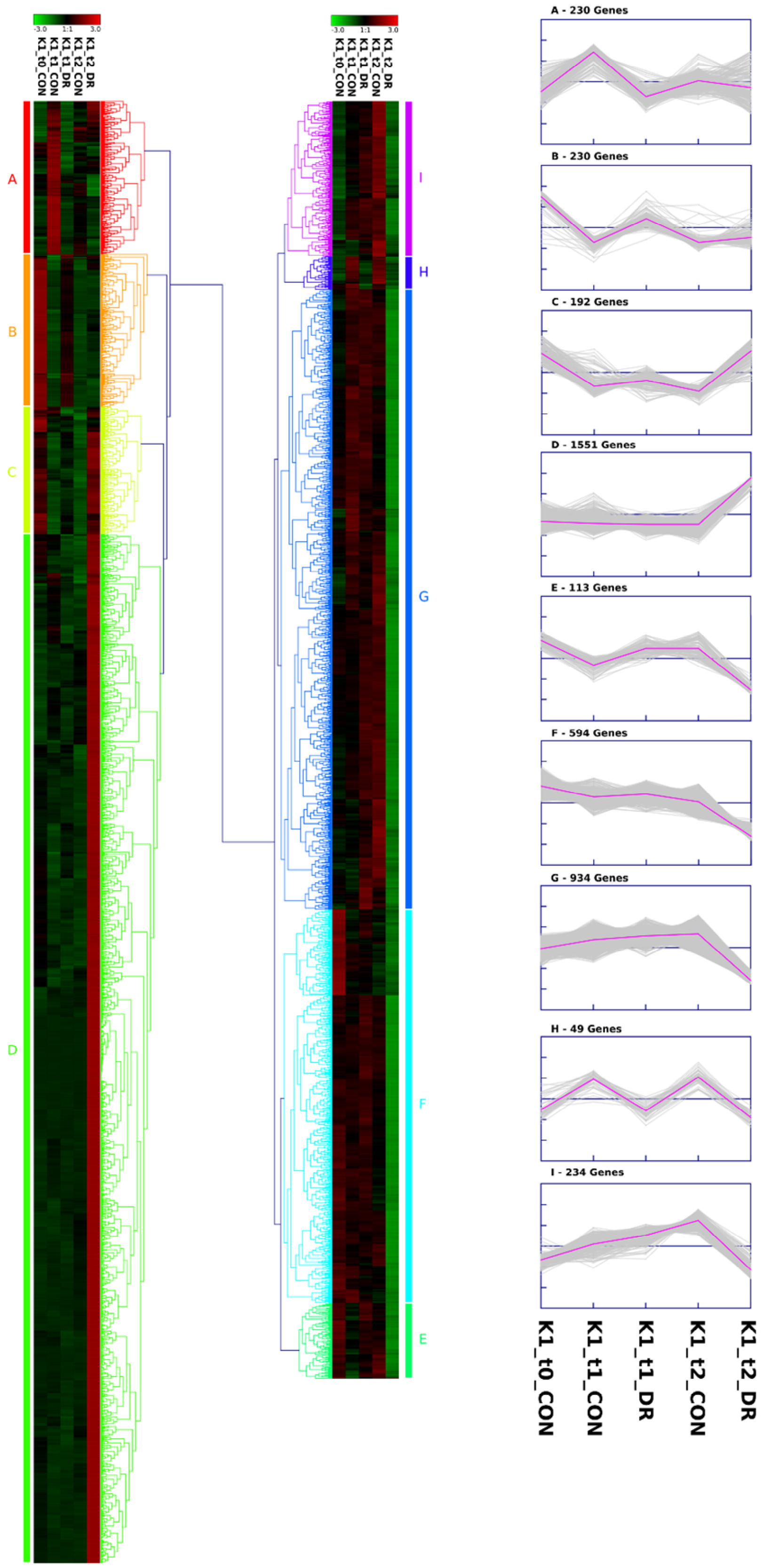

Figure S2. Heat map and cluster results for genes >2 fold differentially expressed between controlled and treated conditions for KOZ1 (4127 genes) at either T1 or T2. Heat map and clustering was created using complete-linkage hierarchical clustering with the Genesis program (Sturn et al., 2002).

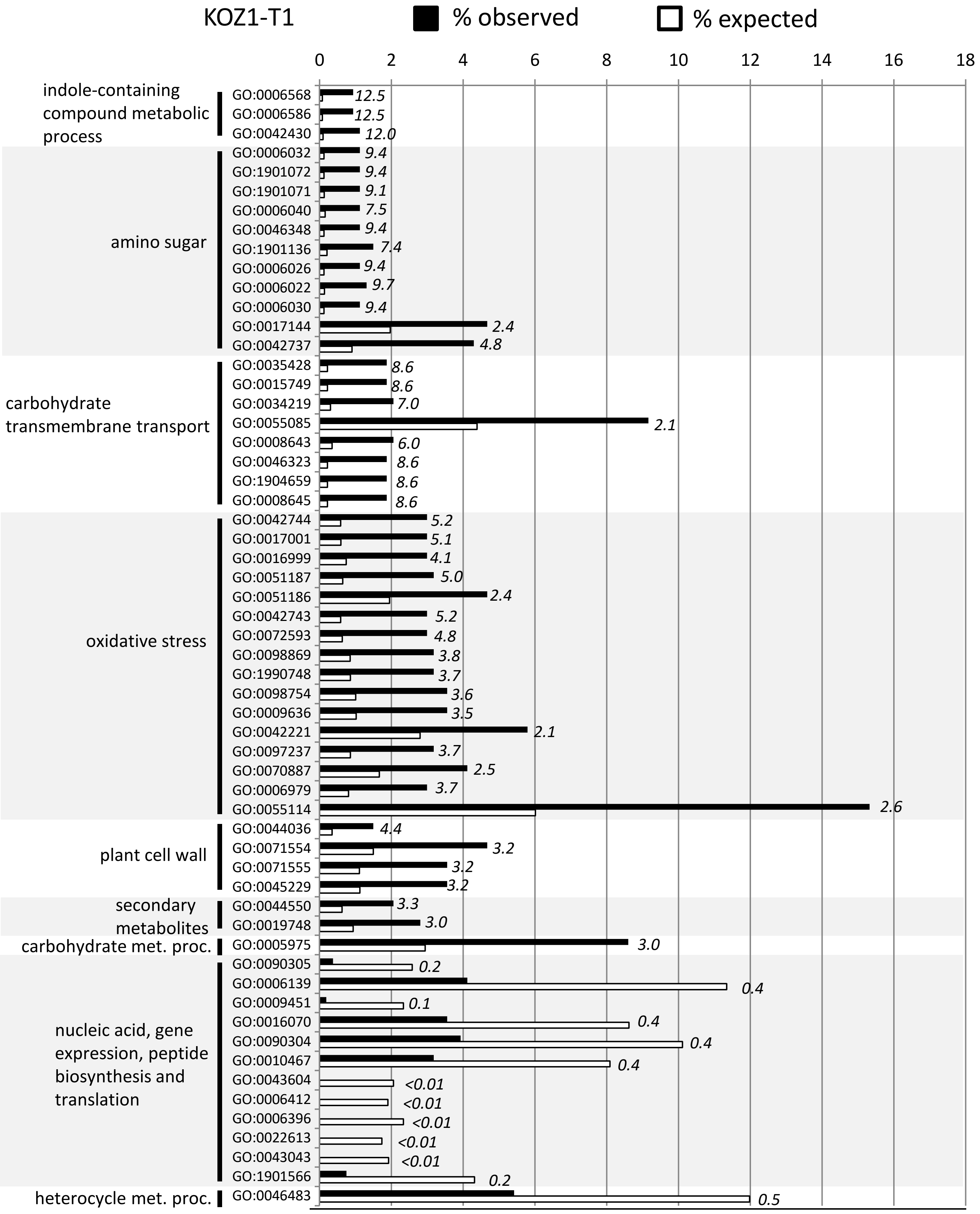

Figure S3. GO term enrichment analysis ($p < 0.05$ FDR corrected) for biological processes of genes >2 fold up or down-regulated in KOZ1-T1. For each GO term, the expected (white bars) and the observed (black bars) percentage is presented. Numbers in italics indicate the fold enrichment. Only GO-terms with more than 2-fold enrichment or depletion are shown.

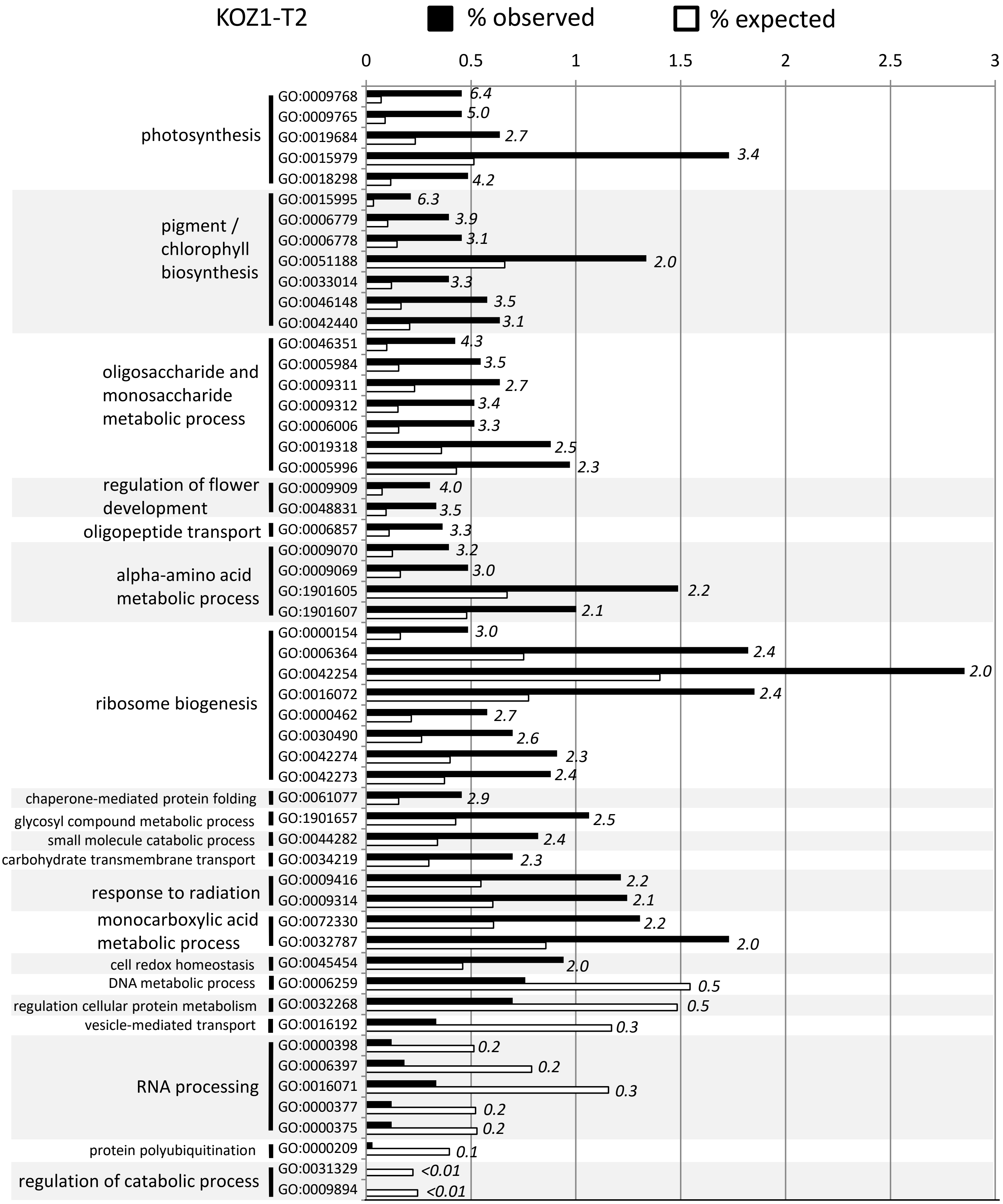

Figure S4. GO term enrichment analysis ($p < 0.05$ FDR corrected) for biological processes of genes >2 fold up or down-regulated in KOZ1-T2. For each GO term, the expected (white bars) and the observed (black bars) percentage is presented. Numbers in italics indicate the fold enrichment. Only GO-terms with more than 2-fold enrichment or depletion are shown.

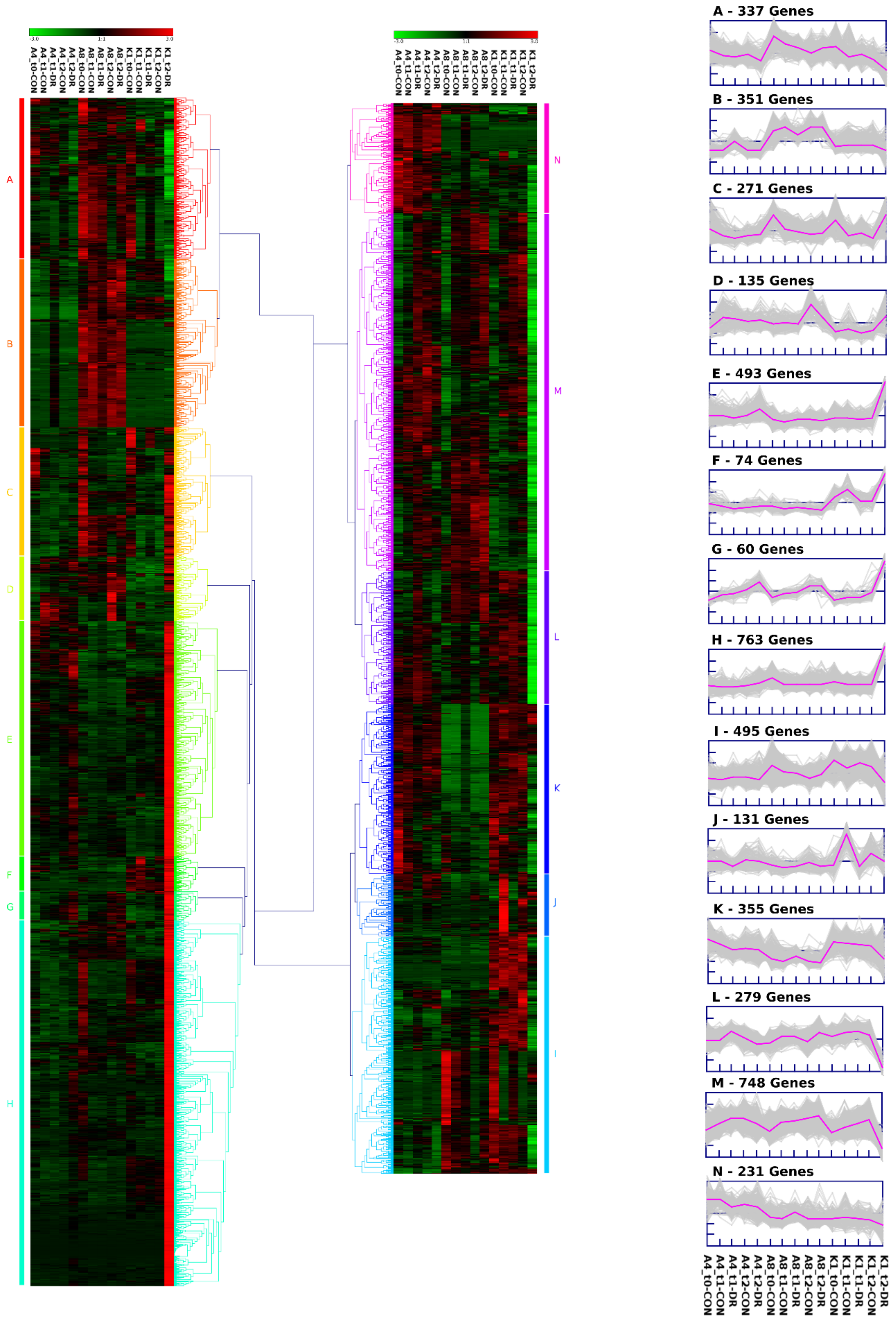

Figure S5. Heat map and cluster results for all of the genes >2 fold differentially expressed between controlled and treated conditions in the drought experiment (total 4723 genes). Heat map and clustering created using complete-linkage hierarchical clustering with the Genesis program (Sturn et al., 2002).

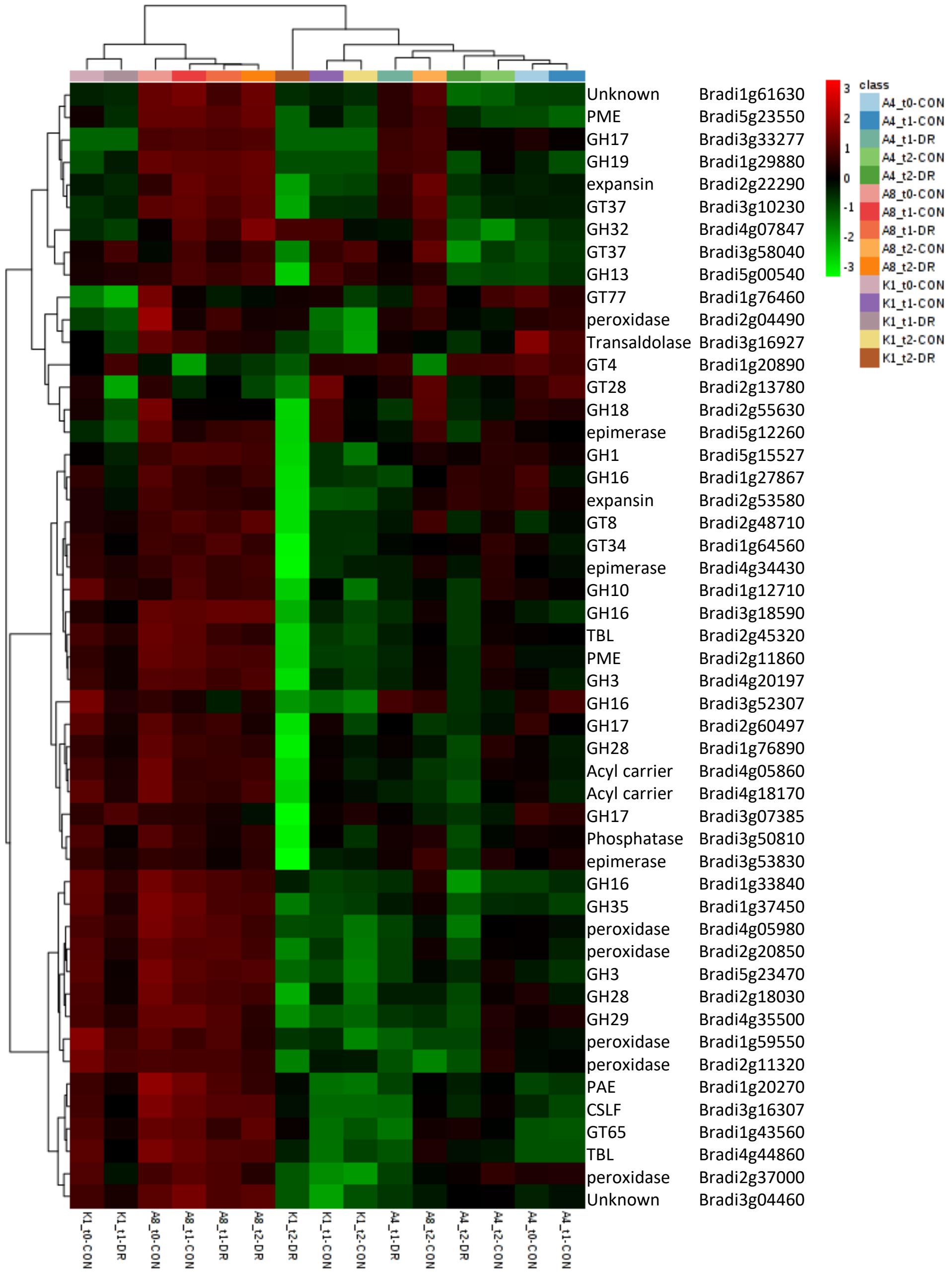

Figure S6. Dendrogram and heat map of the expression levels for 50 genes associated with clusters A+B of the hierarchical cluster analyses for all the genes showing >2-fold differential expression across the samples (see Table S6 for a more detailed description). Abbreviations: A4, ABR4; A8, ABR8; K1, KOZ1; CON, control; DR, drought; GH, glycoside hydrolase; GT, glycosyl transferase; PME, pectin methylesterase; PAE, pectin acetylesterase; TBL, trichome birefringence-like, CSLF, cellulose synthase-like F.

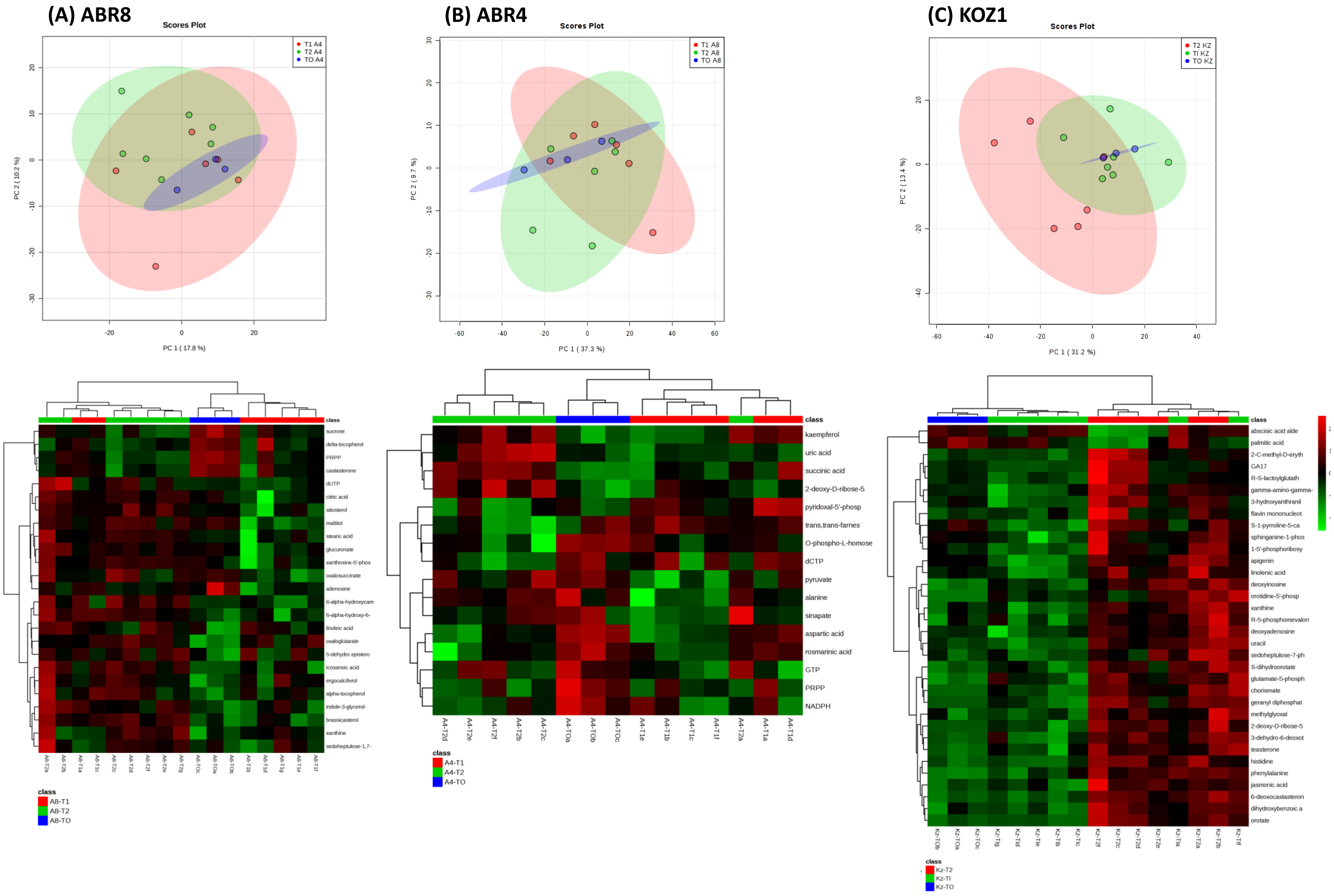

Figure S7. Principal Component Analysis of metabolite profiles in the three *Brachypodium distachyon* genotypes **(A)** ABR8 [A8], **(B)** ABR4 [A4] and **(C)** KOZ1[KZ]) in response to drought stress after 4 days (T1) and 8 days (T2) of with-holding water compared to well-watered controls (T0). Also shown as heat maps are significant metabolite changes (P<0.05; FDR <0.05) for each genotype .

(A) ABR8

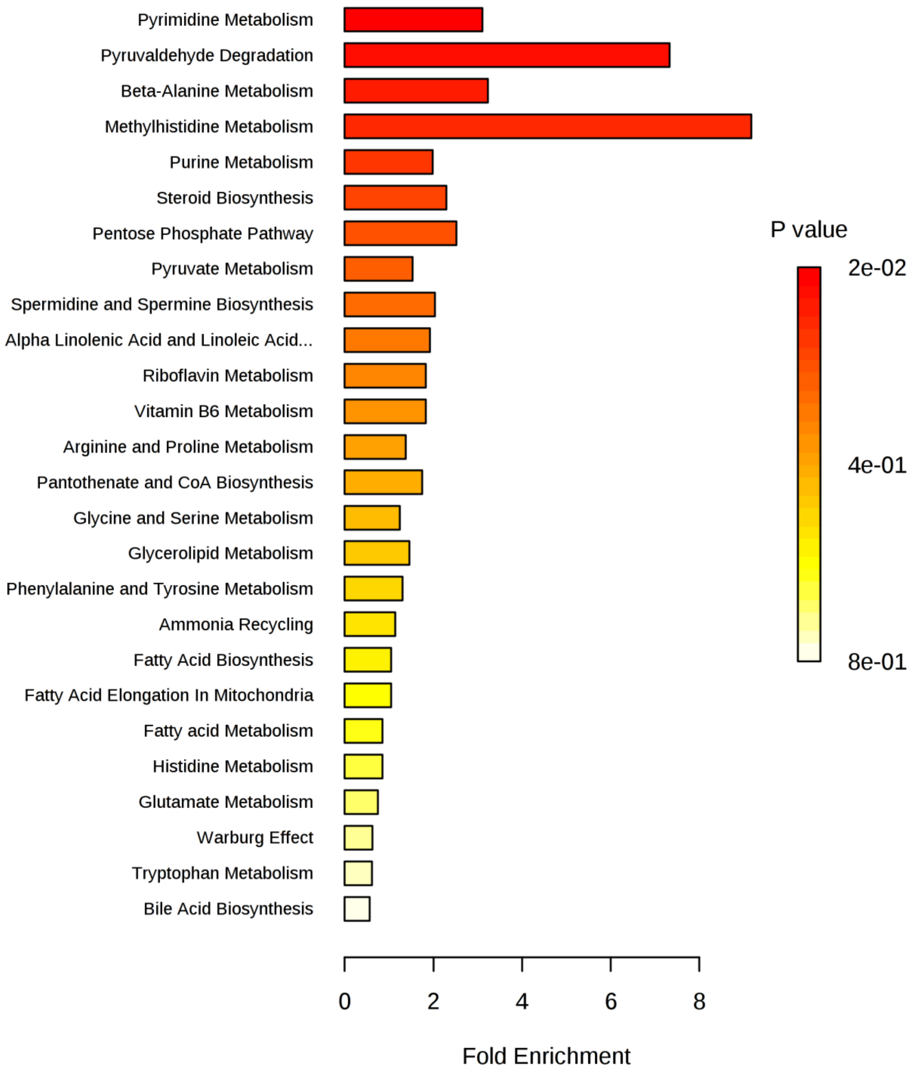

Pathway	Hits	P value	Holm P	FDR
Purine Metabolism	4/74	0.0502	1	1
Starch and Sucrose Metabolism	2/31	0.12	1	1
Glutamate Metabolism	2/49	0.248	1	1
Alpha Linolenic Acid and Linoleic Acid Metabolism	1/19	0.315	1	1

Figure S8. Enriched pathways in the three *Brachypodium distachyon* genotypes **(A)** ABR8 [A8], **(B)** ABR4 [A4] and **(C)** KOZ1[KZ]) in response to drought stress. Tables with significance levels for each genotype are provided for each pathway.

(B) ABR4

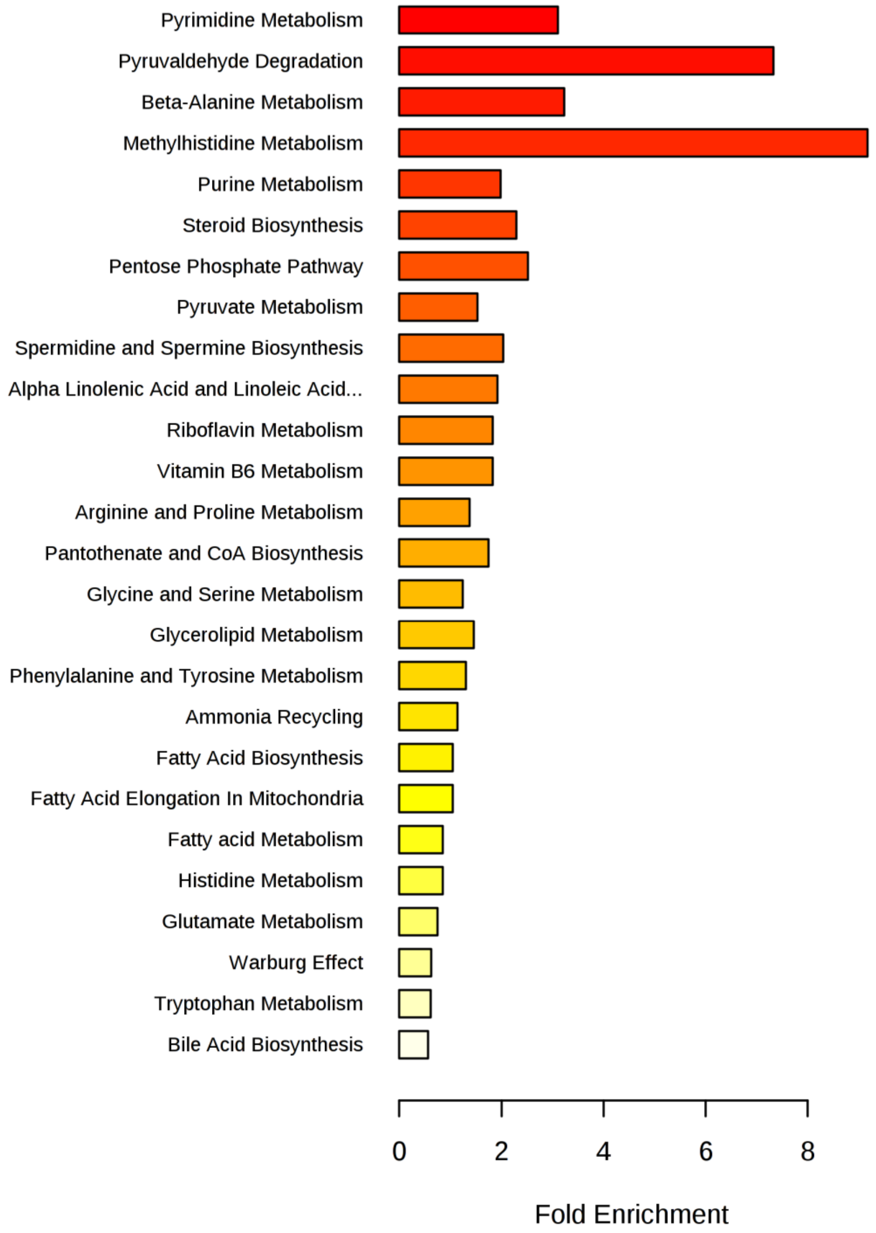

Pathway	Hits	P value	Holm P	FDR
Glutamate Metabolism	7/49	3.07E-06	3.01E-04	3.01E-04
Glucose-Alanine Cycle	4/13	2.62E-05	0.00254	0.00129
Urea Cycle	4/29	7.48E-04	0.0718	0.0244
Alanine Metabolism	3/17	0.00187	0.177	0.0457
Purine Metabolism	5/74	0.00401	0.377	0.0786
Arginine and Proline Metabolism	4/53	0.00733	0.682	0.105
Pentose Phosphate Pathway	3/29	0.00893	0.822	0.105
Malate-Aspartate Shuttle	2/10	0.00958	0.872	0.105
Citric Acid Cycle	3/32	0.0118	1	0.105
Beta-Alanine Metabolism	3/34	0.0139	1	0.114
Aspartate Metabolism	3/35	0.0151	1	0.114
Pyruvate Metabolism	3/48	0.0352	1	0.247
Glutathione Metabolism	2/21	0.0404	1	0.254
Transfer of Acetyl Groups into Mitochondria	2/22	0.0441	1	0.254

(C) KOZ1

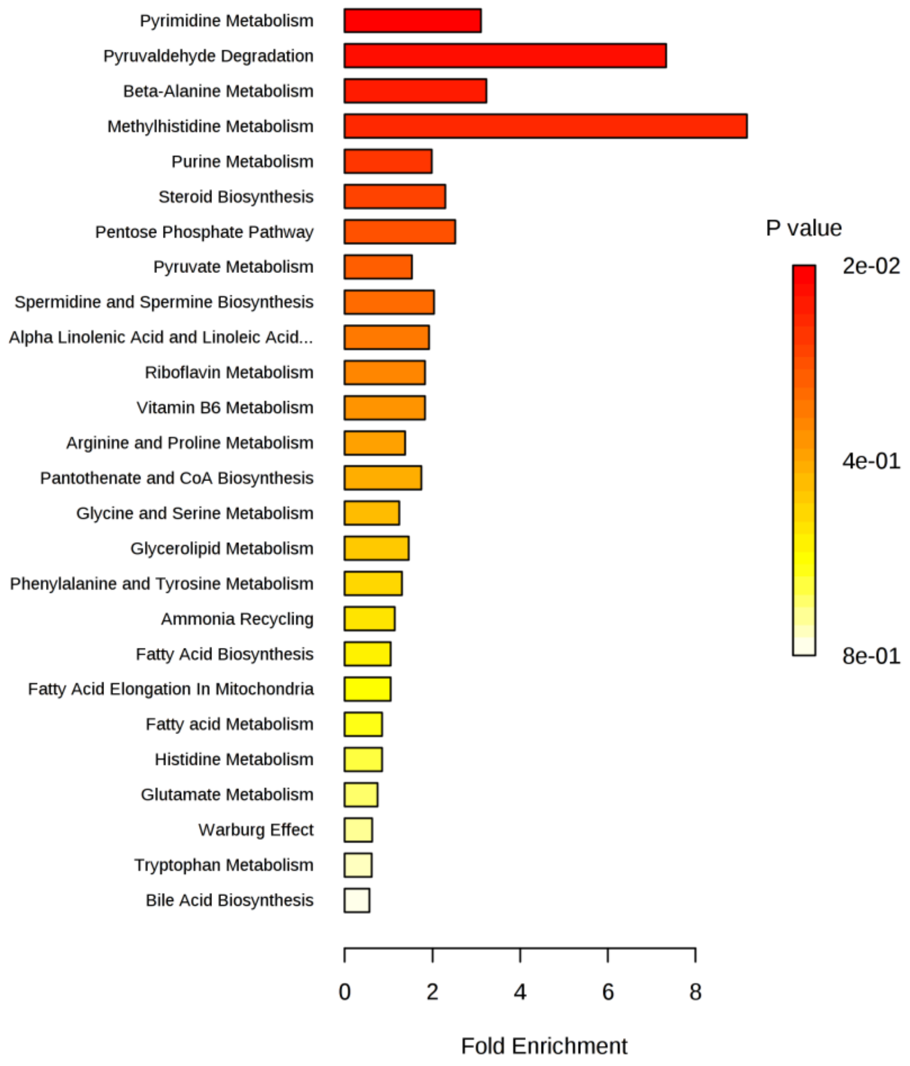

Pathway	Hits	P value	Holm P	FDR
Brassinosteroids	3/9	0.00101	0.08459	3.01E-04
Pyrimidines	4/39	0.01337	1	0.00129
Purines	4/55	0.04222	1	0.0244
